# Activation of Aflatoxin Biosynthesis Alleviates Total ROS in *Aspergillus parasiticus*

**DOI:** 10.3390/toxins10020057

**Published:** 2018-01-29

**Authors:** Gabriel J. Kenne, Phani M. Gummadidala, Mayomi H. Omebeyinje, Ananda M. Mondal, Dominic K. Bett, Sandra McFadden, Sydney Bromfield, Nora Banaszek, Michelle Velez-Martinez, Chandrani Mitra, Isabelle Mikell, Saurabh Chatterjee, Josephine Wee, Anindya Chanda

**Affiliations:** 1 Department of Environmental Health Sciences, University of South Carolina, Columbia, SC 29208, USA; gkenne@email.sc.edu (G.J.K.); pgummadi@email.sc.edu (P.M.G.); mayomi@email.sc.edu (M.H.O.); sdmcfadden2011@gmail.com (S.M.); bromfies@gmail.com (S.B.); banaszen@email.sc.edu (N.B.); velezmar@email.sc.edu (M.V.-M.); cmitra@email.sc.edu (C.M.); immikell@yahoo.com (I.M.); 2 Department of Mathematics and Computer Science, Claflin University, Orangeburg, SC 29115, USA; amondal@claflin.edu (A.M.M.); dbett@claflin.edu (D.K.B.); 3 Environmental Health and Disease Laboratory, Department of Environmental Health Sciences, University of South Carolina, Columbia, SC 29208, USA; schatt@mailbox.sc.edu; 4 Division of Nutritional Sciences, Cornell University, Ithaca, NY 14853, USA; jmw544@cornell.edu

**Keywords:** aflatoxin, aflatoxin biosynthesis, Aspergillus, reactive oxygen species, superoxide dismutase, This work illustrates how aflatoxin biosynthesis contributes to the management of total ROS in *Aspergillus parasiticus*; an established model for studying mycotoxin biosynthesis and secondary metabolism in filamentous fungi. We show that activation of aflatoxin biosynthesis reduces total ROS production in this fungus; which at least in part; is jointly mediated by the aflatoxin pathway regulator; *aflR*; and aflatoxin itself.

## Abstract

An aspect of mycotoxin biosynthesis that remains unclear is its relationship with the cellular management of reactive oxygen species (ROS). Here we conduct a comparative study of the total ROS production in the wild-type strain (SU-1) of the plant pathogen and aflatoxin producer, *Aspergillus parasiticus*, and its mutant strain, AFS10, in which the aflatoxin biosynthesis pathway is blocked by disruption of its pathway regulator, *aflR*. We show that SU-1 demonstrates a significantly faster decrease in total ROS than AFS10 between 24 h to 48 h, a time window within which aflatoxin synthesis is activated and reaches peak levels in SU-1. The impact of aflatoxin synthesis in alleviation of ROS correlated well with the transcriptional activation of five superoxide dismutases (SOD), a group of enzymes that protect cells from elevated levels of a class of ROS, the superoxide radicals (O_2_^−^). Finally, we show that aflatoxin supplementation to AFS10 growth medium results in a significant reduction of total ROS only in 24 h cultures, without resulting in significant changes in SOD gene expression. Our findings show that the activation of aflatoxin biosynthesis in *A. parasiticus* alleviates ROS generation, which in turn, can be both *aflR* dependent and aflatoxin dependent.

## 1. Introduction

Filamentous fungi synthesize and release a diverse array of secondary metabolites into their environment, many of which have profound impacts on agriculture, industry, environmental sustainability, and human health [1]. Many compounds are used as medicines, including statins, penicillin, and other antibiotics. Many others, like aflatoxins and fumonisins, can be life threatening to humans and animals. Aflatoxin B_1_ (AFB_1_), a highly carcinogenic secondary metabolite synthesized by a group of Aspergilli, is a life-threatening toxin causing significant morbidity and mortality worldwide, as well as billions of dollars in annual economic losses [2]. Due to the significant human and agricultural impacts of aflatoxin (AF), its biosynthetic pathway is one of the most characterized and widely studied models for understanding fungal secondary metabolism [3]. 

The aflatoxin biosynthesis process is activated by several environmental cues and orchestrated by a complex regulatory network of more than 25 genes and 17 enzymatic steps [4,5,6,7]. The operation of this network is governed by the interactions of a set of global transcription factors, including LaeA and VeA [4,8,9,10,11,12]. Upon receiving signals from cell surface receptors, these global transcription factors communicate with pathway-specific transcription factors [examples include AflR [13] and GliZ [14,15] to activate specific aflatoxin biosynthesis genes. Many of the enzymes synthesized by this pathway then localize to specific vesicles known as toxisomes [7,16,17,18,19], which provide a platform for the completion of biosynthesis, sequestration, and export of aflatoxin to the environment [7,16,17,18,19]. 

To manipulate secondary metabolism in fungi for the benefit of public and environmental health, it is essential to understand the motivation for a fungal cell to preserve such an energy-consuming metabolic process with enormously complex molecular and cellular organization throughout the course of evolution. One of the most commonly hypothesized functions of fungal secondary metabolites is defense against other organisms in the same ecological niche. Antibacterial properties of secondary metabolites like penicillin and other beta-lactam antibiotics are well established in literature [20]. Beyond antibacterial properties, reports from Rohlfs et al. [21] suggest that aflatoxin and sterigmatocystin protect fungal cells from pests and insects. These studies all suggest that secondary metabolism provides fungi with a survival mechanism in nature. 

Several recent studies suggest that secondary metabolism is integrated with primary metabolism and its associated cellular mechanisms [3,7,22,23], which implies that secondary metabolism may have a regulatory impact on other fungal cellular processes as well. One cellular process that appears to be associated with secondary metabolism in fungi is oxidative stress response. Recently, several basic leucine zipper (bZIP) transcription factors in filamentous fungi have been reported in the literature that not only regulate antioxidant genes participating in oxidative stress response, but are also associated with the regulation of secondary metabolism [3,24,25,26,27,28,29,30,31]. These reports are in line with previous reports [32,33,34,35] suggesting that oxidative stress induces aflatoxin synthesis in *Aspergillus parasiticus*.

While these lines of evidence collectively demonstrate that the two cellular processes (aflatoxin biosynthesis and intracellular oxidative stress management) communicate at different regulatory nodes and are co-regulated, the effect of aflatoxin on oxidative stress remains unclear. In this study we address this knowledge gap through a comparative study of total reactive oxygen species (ROS) output between the wild-type *A. parasiticus* and its mutant, AFS10, in which the aflatoxin pathway regulator gene, *aflR*, is disrupted [36,37]. In addition to measuring ROS, we also conducted a comparative assessment of superoxide dismutase (SOD) gene expression. SODs are conserved in eukaryotes and are synthesized in response to intracellular (O_2_^−^) radicals (a type of ROS) generated as a byproduct of primary cellular functions [38]. To differentiate the aflatoxin-dependent effect on ROS generation from the possible genetic effects (of *aflR* disruption) we also conducted aflatoxin supplementation studies on AFS10. The results of this work provide direct evidence in support of the regulatory role of aflatoxin synthesis on total ROS output and explain the rationale for the co-regulation of oxidative stress with aflatoxin synthesis.

## 2. Results

### 2.1. SU-1 Demonstrates a Significantly Larger Decrease in Total ROS Compared to AFS10 between 24 h and 48 h

**Figure 1:**
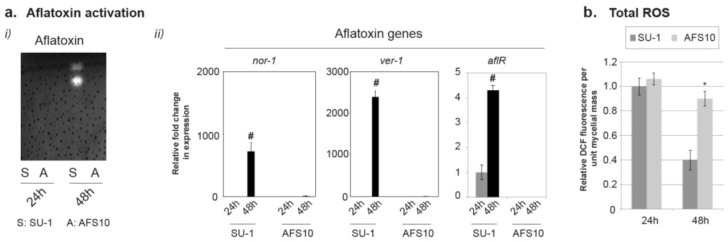
Decrease of total ROS during activation of aflatoxin biosynthesis. (**a**) Comparison of (i) aflatoxin accumulation and (ii) Gene expression levels relative to 24 h of three aflatoxin pathway genes in SU-1 and AFS10. (**b**) Comparison of total ROS at 24 h and 48 h. The error-bars represent standard error of the mean. The two-tailed *p*-value was determined using unpaired *t*-test (GraphPad statistical software). #, Significant difference of transcript levels between 24 h and 48 h (*p*-value < 0.05, *n* = 3); * Significant difference of total ROS between SU-1 and AFS10 (*p*-value < 0.05, *n* = 3).

Aflatoxin biosynthesis is activated in SU-1 at 24 h under our culture conditions and reaches peak levels by the start of the stationary phase at 48 h [7,39]. Under these conditions aflatoxin biosynthesis is not activated in AFS10. As shown in Figure 1a, during the 24 h–48 h-time window, aflatoxin accumulation in the growth medium was observed and aflatoxin genes were activated in SU-1 but not in AFS10. The genes *nor-1* and *ver-1* were chosen as representative aflatoxin genes that demonstrated drastic increases in expression similar to previously reported semi-quantitative analysis of transcript and protein analysis [40]. Quantitative comparison of total ROS (Dichlorodihydrofluorescein [DCF] fluorescence measurements shown in Figure 1b) shows that at 24 h both strains demonstrate similar levels of total ROS, but by 48 h the total ROS decreased at a significantly higher rate in SU-1 than in AFS10. This demonstrated an association between the activation of aflatoxin biosynthesis and a decrease in total ROS, which may be attributable to either the presence of aflatoxin or the regulatory role of *aflR*.

### 2.2. Higher Total ROS in AFS10 Compared to SU-1 at 48 h Associates with Significant Differences in SOD Gene Expression

#### 2.2.1. Bioinformatics Analyses of SOD Genes

Since SOD genes are synthesized in eukaryotes in response to intracellular O_2_^−^ radicals (a type of ROS) generated as a byproduct of primary cellular functions [38], we investigated whether higher ROS at 48 h in AFS10 is correlated with the transcriptional activation of SOD genes. As a first step to do so we initiated a search for SOD genes within the available genome database of a closely related species, *A. flavus* [41] and identified five amino acid sequences (Table 1). Out of these five sequences, two different sequences of copper–zinc SOD genes are annotated in the database as CuZn*sod*1 and cytosolic CuZn*sod*, two sequences of iron SOD are annotated as Fe*sod* and Fe*sodA*, and one manganese SOD is annotated as Mn*sod*. These five sequences were queried against the PROSITE database [42] to verify whether they contained any of the conserved functional domains or patterns that are present in the well-characterized SODs within the database. 

As shown in Table 2A, two of these sequences contained superoxide dismutase (SOD) signatures. CuZn*sod*1 had two typical CuZn SOD signatures. The conserved sequence (AFHVHQfGDnT) matched with the consensus pattern, [GA]-[IMFAT]-H-[LIVF]-H-[S]-x-[GP]-[SDG]-x-[STAGDE], for signature 1 where 2 *H*’s are copper ligands. Similarly, conserved sequence (GNAGaRpACgvI) matched with the consensus pattern, G-[GNHD]-[SGA]-[GR]-x-R-x-[SGAWRV]-C-x(2)-[IV], for signature 2, where *C* is involved in a disulfide bond. Mn*sod* contained the conserved sequence, DmWEHAYY, corresponding to manganese and iron SOD signature. This signature matched with the consensus pattern, D-x-[WF]-E-H-[STA]-[FY](2), where *D* and *H* are manganese/iron ligands.

The PROSITE database was then used to investigate whether the three other sequences that did not contain typical SOD motifs contained regions that have high probability of occurrence (frequent patterns) in SODs. The remaining three amino acid sequences displayed the four patterns (an *N*-myristoylation site, a Casein kinase II phosphorylation site, and *N*-glycosylation site, and a Protein kinase C phosphorylation site) that are the most frequently present within the 390 SOD genes available in PROSITE database, suggesting strongly that these are SOD sequences (Table 2B).

#### 2.2.2. Expression Profiles of SOD Genes

**Figure 2:**
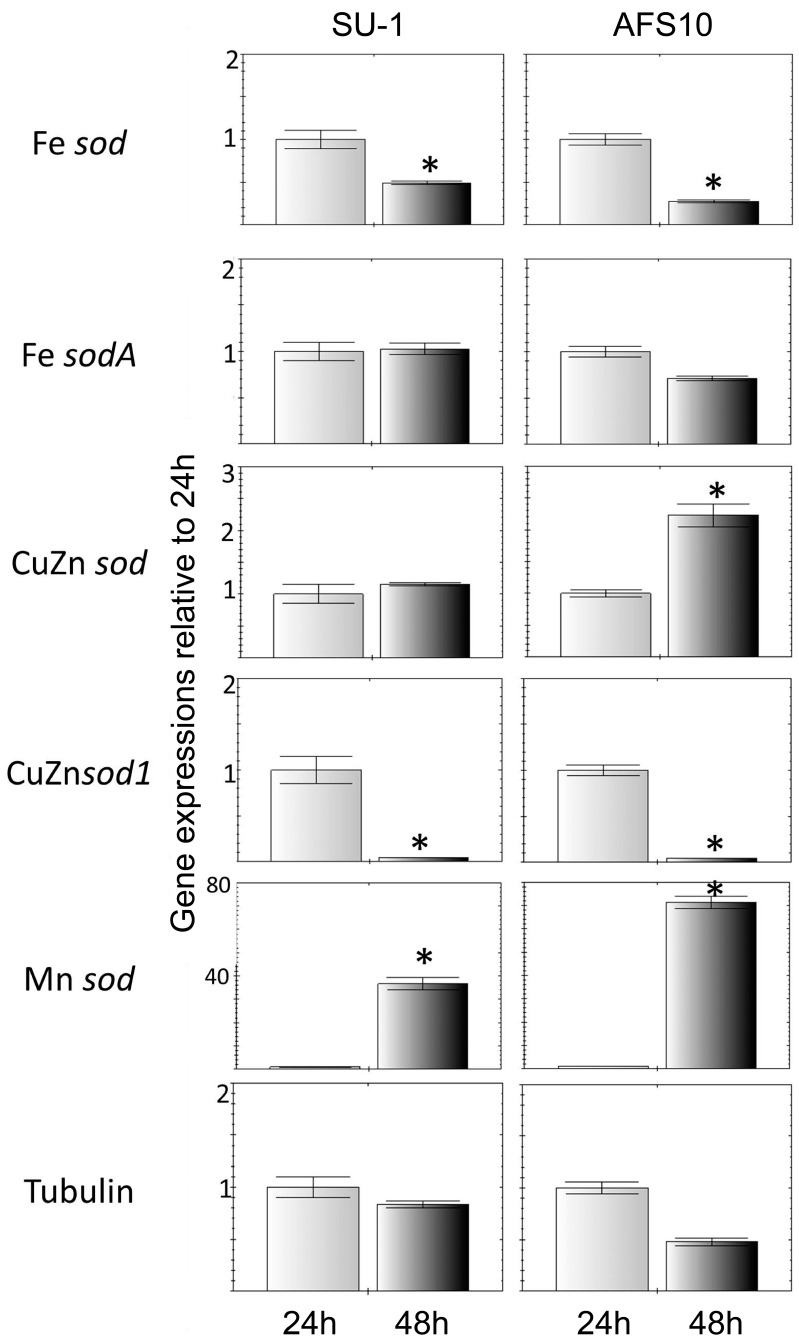
Comparison of SOD gene expression in SU-1 and AFS10. Quantitative PCR (qPCR) comparison of SOD gene expression in the two strains at 24 and 48 h of culture growth. All expression quantifications were conducted in triplicate. For each gene the expression value was normalized against the 18s rRNA reference gene and compared to a β-tubulin control. The expression values for each target gene at early stationary phase (48 h) were expressed as the fold change relative to 24 h time point. Fold changes ≥2.0 were considered up- or down-regulated. All data and statistical analysis (Student’s *t*-test) were performed using CFX Manager software (Bio-Rad Laboratories). Compared to 24 h gene expression, Fe*sod* showed a significant decrease in both the wild-type (2.1-fold; *p* = 0.003) and AFS10 (3.9-fold; *p* < 0.001); Fe*sodA* showed no significant change for either strain; CuZn*sod* expression did not change in the WT, but showed a 2.1-fold increase (*p* = 0.003) in AFS10; CuZn*sod*1 showed a large, significant decrease in expression for both the WT (22.4-fold; *p* = 0.001) and AFS10 (26.4-fold; *p* < 0.001); Mn*sod* had a dramatically significant 36.2-fold increase in gene expression in the WT (*p* < 0.001), and an even greater 69.8-fold increase in AFS10 (*p* < 0.001) compared 24 h expression. (Raw gene expression data is included as Supplementary Figure S1). * Indicates statistically significant difference from respective 24 h gene expression; *p* ≤ 0.05.

The gene expression of all five SODs was examined in both SU-1 and AFS10 at 24 h and 48 h post-inoculation in yeast extract sucrose (YES). Quantitative comparison of the transcript levels between 24 h and 48 h, with levels normalized to 24 h (raw expression data relative to 18s rRNA shown in Figure S1) are shown in Figure 2 and the list of primers used are mentioned shown under Table 3. The data suggest that SOD expression profile in this fungus is growth phase dependent. Hence, while the expressions of Fe*sod* and CuZn*sod*1 are higher in 24 h cultures (corresponding to the exponential growth phase) the Mn*sod* expression is significantly higher in the 48 h cultures (corresponding to the stationary growth phase). As seen in Figure 2, AFS10 displayed a significantly larger increase in Mn*sod* expression from 24 h to 48 h (~70-fold increase in AFS10 versus a ~40 fold increase in SU-1). Additionally, CuZn*sod* expression that remained constant in SU-1 showed a significant increase from 24 to 48 h in AFS10. No significant difference was observed between SU-1 and AFS10 for genes Fe*sod* and CuZn*sod*1. Our results, therefore, demonstrate an association between higher ROS levels in AFS10 (compared to SU-1) and absence of aflatoxin biosynthesis during the 24 h–48 h time window in AFS10 with the significantly larger increases (compared to SU-1) in Mn*sod* and CuZn*sod* transcripts from 24 h to 48 h. 

### 2.3. Aflatoxin Supplementation to AFS10 Growth Medium Changes Total ROS Output without Changing the SOD Transcript Levels

**Figure 3:**
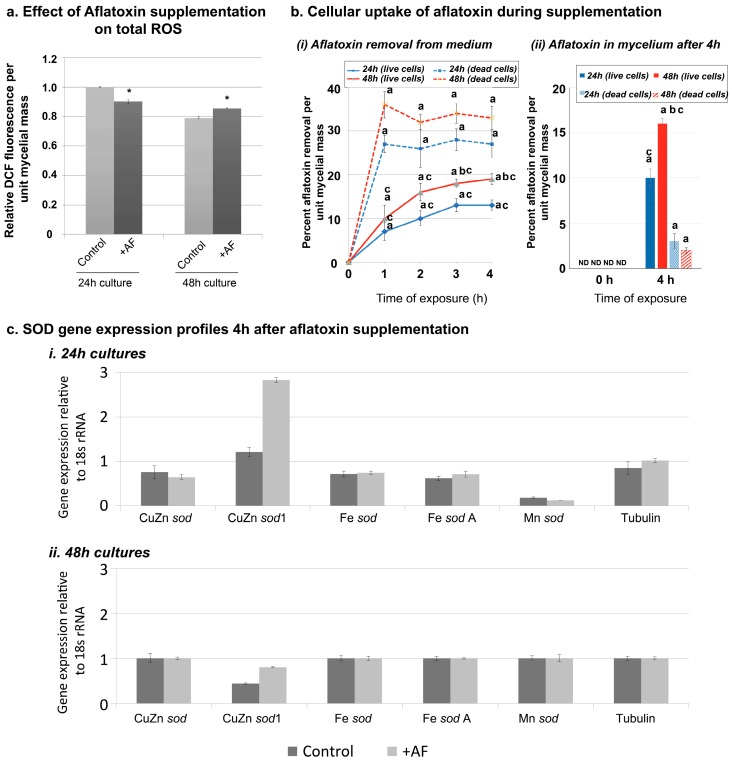
Aflatoxin supplementation to AFS10. (**a**) Effect on total ROS. A quantitative comparison of ROS in AFS10 supplemented with 50 ppm aflatoxin (in 70% methanol) and a 70% methanol control was conducted. Total ROS was quantified at 24 h and 48 h of growth + 4 h of incubation in 1 μM 2′,7′-dichlorofluorescein diacetate (DCFH-DA) in phosphate buffered saline (PBS) substrate with the corresponding AF concentration. Error-bars represent SEM. (*) denotes statistically significant difference (*p* < 0.05; *n* = 3) in ROS compared to the 70% methanol control for the corresponding growth time. (**b**) Cellular uptake of aflatoxin during aflatoxin supplementation. (i) Percent removal of aflatoxin from the supplementation medium in live cells of 24 h and 48 h AFS10. The percent removal was calculated at every hour until 4 h to compare the aflatoxin removal pattern by live cells with the dead cells that allow free diffusion from the medium into the cells. (ii) Percent aflatoxin accumulation in the mycelium of 24 h and 48 h cultures. Aflatoxin in the mycelia of live cells was compared to the dead cells. Error-bars represent SEM. a, statistically significant difference (*p* < 0.05; *n* = 3) in aflatoxin levels with 0 h, b, statistically significant difference (*p* < 0.05; *n* = 3) in aflatoxin levels between 24 h and 48 h cultures, c, statistically significant difference (*p* < 0.05; *n* = 3) in aflatoxin levels between live and dead cells at a particular time-point. (**c**) Comparison of SOD gene expression in aflatoxin supplemented and control AFS10. qPCR comparison of SOD gene expression in the control and 4 h aflatoxin supplemented cells. The gene expression values were normalized against the 18s rRNA reference gene. Fold changes ≥2.0 were considered up- or down-regulated. All data and statistical analysis (Student’s *t*-test) were performed using CFX Manager software (Bio-Rad Laboratories).

The significantly larger decrease in total ROS in SU-1 compared to AFS10 could either be aflatoxin dependent, *aflR* dependent, or both. To examine if total ROS production is in-part aflatoxin dependent, we investigated whether aflatoxin supplementation to AFS10 impacts the total ROS levels. The results from this experiment are shown in Figure 3. A 4 h supplementation of 24 h mycelia with total aflatoxin isolated from an SU-1 growth medium resulted in a significant decrease of total ROS (Figure 3a). In contrast, the 4 h aflatoxin supplementation to 48 h AFS10 mycelia significantly increased the total ROS. To understand this differential effect of aflatoxin supplementation on the 24 h and 48 h AFS10 cultures, we conducted an examination of aflatoxin uptake by the mycelium during the 4 h time-period. As shown in Figure 3b, the percentage removal of aflatoxin per unit mass of mycelium by the end of 4 h was significantly higher for 48 h cultures than 24 h cultures. This data also agreed with the aflatoxin accumulation in the mycelia, which demonstrated a significantly higher accumulation of aflatoxin in 48 h cultures than in 24 h cultures. To examine whether the aflatoxin accumulation was a free diffusion versus an active uptake mechanism by the mycelium, we conducted a similar experiment with equal masses of dead AFS10 cultures obtained upon autoclaving the cultures. Our results demonstrate that while the free diffusion of aflatoxin from the medium to the immersed dead cells resulted in a faster removal of aflatoxin from the medium, the aflatoxin could not be retained in the dead mycelia unlike the live cells, when taken out of the medium and washed. Collectively the gradual increase in aflatoxin removal from the medium (unlike the dead cells) and the ability of retaining the aflatoxin in the mycelium suggests an active uptake mechanism of aflatoxin by the cells. The significantly higher uptake of aflatoxin in 48 h cultures than the 24 h cultures suggest that the differential effects of the aflatoxin supplementation on total ROS in the 24 h versus 48 h cultures are associated with the differential levels of aflatoxin uptake by the mycelia of these ages.

Finally, we also examined whether aflatoxin supplementation resulted in changes in the expression levels of the SOD genes either in 24 h or 48 h cultures. Contrary to the total ROS readings, there were no significant changes in SOD expression that were attributable to AF supplementation (Figure 3c), thereby suggesting the possibility that aflatoxin supplementation induced changes in the total ROS are acute biochemical effects.

## 3. Discussion

**Figure 4:**
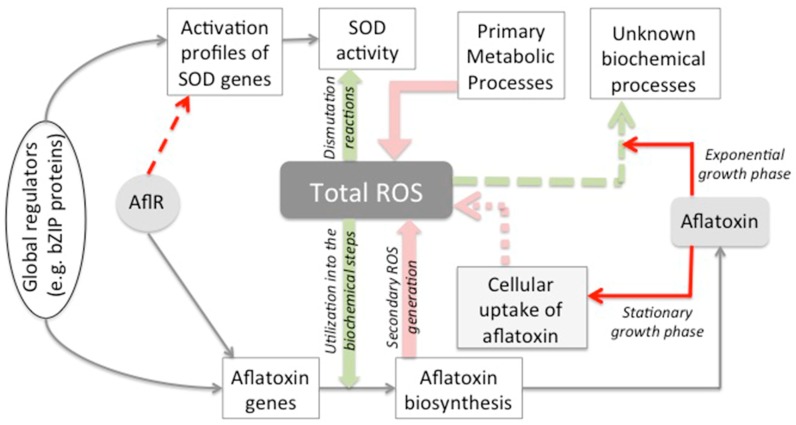
Proposed model for total ROS management in *A. parasiticus*. Based on our current findings and previous reports we propose that aflatoxin-dependent protection occurs in one or a combination of the following ways: (a) utilization of ROS in the biochemical steps of the biosynthesis pathway [33], (b) aflatoxin-dependent reduction of ROS in cells at exponential growth phase (current study) and (c) *aflR*-dependent reduction of ROS (current study) possibly through its gene regulatory impacts outside the aflatoxin pathway gene cluster [43,44]. Aflatoxin-dependent biochemical processes that sequester ROS still remain uncharacterized (green dashed arrow). Pink arrows indicate the sources of ROS accumulation. These include ROS generation from primary metabolic processes, secondary ROS generated from aflatoxin biosynthesis [45], and ROS generated upon aflatoxin uptake by cells during stationary phase of growth (based on aflatoxin supplementation data from 48 h AFS10 cultures in the current study). The mechanisms that result in ROS accumulation upon cellular uptake of aflatoxin remains uncharacterized (pink dashed arrow). The model can now explain the physiological need of the cells to co-regulate secondary metabolism (in this case, aflatoxin biosynthesis) and oxidative stress response through the bZIP proteins [3,24,25,26,27,28,29,30,31]. Red arrows indicate the contributions of the current study. The molecular mechanism of *aflR*-mediated regulation of SOD genes remains uncharacterized (red dashed arrow) and will be investigated in our follow up studies.

This study provides the first direct demonstration of the regulatory role of a secondary metabolite on a cellular process of the producer’s oxidative stress management. It also can now explain the previous reports on the cross-talk between oxidative stress and secondary metabolism [32,33,34,35]. Based on our current findings and previously published literature, we propose here a ROS management model for aflatoxin producers (illustrated in Figure 4). According to this model, aflatoxin biosynthesis protects cells against ROS accumulation from at least three different sources: (a) primary metabolic processes, (b) secondary ROS generated from aflatoxin biosynthesis, as proposed previously by Roze et al. [45], and (c) ROS generated upon aflatoxin uptake by cells during the stationary phase of growth (aflatoxin supplementation data from 48 h AFS10 cultures in the current study). The aflatoxin-dependent protection occurs in one or a combination of the following ways: (a) utilization of ROS in the biochemical steps of the biosynthesis pathway [33], (b) aflatoxin-dependent reduction of ROS in cells at exponential growth phase (aflatoxin supplementation data from 24 h AFS10 cultures in the current study) and (c) *aflR*-dependent reduction of ROS (current study) possibly through its gene regulatory impacts outside the aflatoxin pathway gene cluster [43,44]. Our data support the likelihood that disruption of *aflR* blocks all the three modes of aflatoxin-dependent protection, leading to a higher accumulation of super-oxide radicals in AFS10 compared to SU-1. This can explain the increased demand for SOD activation and the higher SOD transcript levels in AFS10 than in SU-1.

To address the direct effect of aflatoxin on total ROS, we designed a 4 h supplementation experiment to compare the individual effects of the supplementation on the 24 h and the 48 h AFS10 cultures. We understand based on previous literature [7,40,46] that 24 h cultures and 48 h cultures (under our standard growth conditions), are very different physiological systems; 24 h cultures demonstrate no secondary metabolite synthesis and in 48 h cultures secondary metabolite synthesis occurs at peak levels. The 4 h time was optimized from initial uptake experiments in which we noticed no significant increase in the growth of the mycelia until 4 h under the given experimental conditions (data not shown). We reasoned that supplementation beyond 4 h would result in adaptation of fungal cells and that would not allow us to observe the acute effects as described in this study.

It is speculated that fungal toxisomes, which are sites for the synthesis and compartmentalization of secondary metabolites [7,17], receive input from peroxisomes and mitochondria as well as from the secretory and Cvt vesicle transport pathways [17]. A significant increase in the mitochondrial SOD, MnSOD, at 48 h suggests that it is primarily responsible for dismutating the superoxides during the stationary phase. Previous proteomic data on fungal toxisomes in *A. parasiticus* [22] demonstrated an enrichment of superoxide dismutases, especially MnSOD, within the toxisomes as well. Catalases also present in the tosixomes then convert the hydrogen peroxide product of the dismutation reactions into oxygen and water. The data shown here correspond increased MnSOD with ROS levels after the initiation of aflatoxin biosynthesis support the possibility that superoxides are compartmentalized into fungal toxisomes in addition to the mitochondria, and become available for incorporation into secondary metabolite biosynthetic pathways, including aflatoxin synthesis, in addition to dismutation by SODs. We emphasize here that while the SOD expression profiles are closely and independently associated with total ROS and the activation of aflatoxin biosynthesis, our data (Figure 3c) do not support aflatoxin as a direct regulator of SOD gene transcription, thereby suggesting that additional regulator(s) work in concert with AflR to regulate SOD gene expression. An example of such a regulator is the bZIP transcription factor AtfB [3,47], which is in part one regulator of the SODs and the cellular response to intracellular oxidative stress [25,26,47] that binds to *aflR* gene promoter and physically interacts with the AflR [48,49]. 

One limitation of this study is the lack of an appropriate methodology for clean biochemical measurements specific for superoxide radicals (O_2_^−^) within Aspergillus cells. Commercially available small molecules like DMPO, that can successfully trap O_2_^−^ within mammalian and yeast cells, have conventionally been used for such O_2_^−^ quantifications. However, these small molecules fail to enter Aspergillus cells (data not shown). Within the cell, toxisomes are very dynamic systems that are continuously exporting protein and metabolite contents to the extracellular environment [16], at which time any present superoxide radicals would be detectable by molecules such as DMPO. Therefore, unless the extremely unstable O_2_^−^ radicals are incorporated into the location of aflatoxin synthesis within toxisomes, as in case of SU-1 (but not in AFS10), commercial cellular stains like MitoSOX or CellROX cannot provide a true overall quantification of the total O_2_^−^ radicals or total ROS through cellular imaging experiments as done for many mammalian cells, and will lead to inaccurate interpretations. The protocol used in these experiments is based on a methodology previously established by Chang et al. [50]. The method allows the substrate DCFH-DA to react with the total ROS generated within mycelia and form the fluorescent marker DCF that can then be quantified spectrophotometrically. While we acknowledge the technical limitations of the DCFH-DA probe in providing an accurate quantification of superoxides and total ROS [51], we reason that our experimental design, being dependent of relative ROS levels rather than accurate ROS quantifications, was able to circumvent these challenges and therefore our interpretations on relative ROS levels were not impacted. 

In conclusion, our findings establish the foundation for a long-term study that will investigate the molecular, cellular, and biochemical mechanisms underlying the differential effects of aflatoxin on ROS accumulation in cells that are in an exponential growth phase versus those in a stationary phase. We hypothesize based on these findings that secondary metabolites have a regulatory role in the cellular coordination of secondary metabolism and oxidative stress response in filamentous fungi. Our future studies will shed more light on revealing the complexity of such coordination and thereby help identify novel targets for the manipulation of secondary metabolism.

## 4. Materials and Methods

### 4.1. Strains, Media, and Growth Conditions

*Aspergillus parasiticus* wild type strain SU-1 (ATCC56775) and the *aflR* dustrupted mutant, AFS10 [37,40], were used for this study. Yeast extract sucrose (YES) (2% yeast extract, 6% sucrose; pH 5.8) was used as the liquid growth medium for the entire study for both strains. Fungal cells were grown for 24 h and 48 h by inoculating 10^7^ spores per 100 mL of growth medium and incubating the cells at 29 °C in a dark orbital shaker at 150 rpm. 

### 4.2. Quantification of ROS

Comparison of ROS concentrations between SU-1 and AFS10 was conducted spectrophotometrically using 2′,7′-dichlorofluorescein diacetate (DCFH-DA) based on a previously described protocol [50]. Equal weight (0.5 g) of mycelia from a 24 and 48 h culture was placed into 1 mL of freshly made 1 μM DCFH-DA in phosphate buffered saline (PBS). After 4 h of incubation in the dark at room temperature (25 °C), the fluorescent yield of the DCFH-DA oxidation product, dichlorofluorescin (DCF), was measured using a Victor™ X3 2030 Multilabel Reader (PerkinElmer, Waltham, MA, USA) with an excitation/emission wavelength of 490/525 nm.

### 4.3. Identification of Superoxide Dismutase Genes

Since functional characterization of the SOD genes in *A. parasiticus* has not yet been completed, a bioinformatics analysis was performed to identify SOD gene sequences to allow for a comparative expression analyses to address our hypothesis. The SOD genes analyzed in this study were identified by searching for “superoxide dismutase” in the accessible genome database [41] of *A. flavus*, a close relative of *A. parasiticus* that exhibits ~98–100% amino-acid sequence identity with *A. parasiticus* proteins that have been sequenced [3]. The search rendered five annotated amino-acid sequences which were then queried in the PROSITE database [42] against the 390 available SOD genes to investigate whether they contained (a) the conserved functional domains typical of SODs, or (b) motifs with a high probability of occurrence that are commonly present in the SOD genes. Details of these sequences and queries can be found in Table 1. 

### 4.4. RNA Extraction, Purification and cDNA Synthesis

Total RNA was extracted from cells harvested using a TRIzol-based (TRIzol Reagent; Invitrogen, Carlsbad, CA, USA) method previously described [7]. Within 24 h of extraction, RNA cleanup was performed using a Qiagen RNEasy Cleanup Kit (Qiagen, Valencia, CA, USA), and samples were stored at −80 °C. Total RNA was then reverse transcribed to cDNA using iScript™ cDNA Synthesis Kit (BioRad Laboratories, Hercules, CA, USA). All samples were checked for concentration and purity after each step using a NanoDrop 2000 Spectrophotometer (Thermo-Fisher Scientific, Waltham, MA, USA). All cDNA samples were stored at −20 °C until subsequent PCR quantification.

### 4.5. Quantitative PCR Assays

Expression of SOD genes was examined by quantitative PCR assays (qPCR) using SsoAdvanced Universal SYBR Green Supermix (BioRad Laboratories, Hercules, CA, USA) and gene specific forward and reverse primers (Table 2) designed using Primer3 online software [52]. Reactions were performed per BioRad SYBR Green protocol guidelines and quantified using a CFX96 thermal cycler (Bio-Rad Laboratories, Hercules, CA, USA).

The 18s ribosomal DNA was used as a reference in the gene expression experiments, with β-tubulin used as a positive control rather than a reference gene. This use of β-tubulin in this manner provided proof of consistent quantification across all experiments and revealed an expected range of variation within the protocol. Expression of each SOD gene was obtained from the threshold cycle values normalized against 18s rDNA in each sample. All RT-PCRs were performed in triplicate for each gene per sample. For quantitative comparison of gene expression, the expression values for each target gene at the early stationary phase (48 h) were expressed as the fold change relative to the 24 h time point to reflect changes associated with the initiation of aflatoxin biosynthesis, which begins at 30 h [45]. All data analysis was performed using CFX Manager software (Version 3.1, Bio-Rad Laboratories, Hercules, CA, USA, 2012).

### 4.6. Aflatoxin Supplementation Experiments

For aflatoxin supplementation studies, 0.5 g of AFS10 mycelia were collected from YES media at 24 and 48 h and each placed in 12-well trays containing 1 mL of their culture media. Total aflatoxin (in 70% methanol solution) isolated from an SU-1 culture using our standard chloroform-methanol isolation procedure [53] was added to each sample well at a final concentration of 50 ppm. The control mycelia were supplemented with an equal volume of 70% methanol solution. After a 4 h incubation, mycelia were transferred to 1 mL of 1 μM DCFH-DA in PBS substrate for an additional 1 h incubation in the dark before being measured (in triplicate) for DCF fluorescence. Aflatoxin uptake into the mycelia during the incubation period was quantified by measuring total percent removal of aflatoxin from the medium every hour until 4 h and by measuring the total accumulation of aflatoxin in the mycelium in parallel, after 4 h. Percent removal of aflatoxin from the medium was calculated as follows: Percent removal = ((Initial total aflatoxin in the supplementation medium − total aflatoxin in the medium at a time point)/Initial total aflatoxin in the supplementation medium) × 100. Aflatoxin was quantified in the medium as described below. Aflatoxin accumulation in the mycelium was measured after washing three times with PBS buffer followed by extracting aflatoxin from the mycelium using a chloroform: methanol procedure as described previously [40]. Aflatoxin in the extract was then measured using an enzyme-linked immunosorbent assay (ELISA). Dead cells of AFS10 obtained upon autoclaving at 121 °C for 15 min were used in the uptake experiments as controls for free diffusion systems. Loss of viability in these cells was confirmed prior to experimentation by confirming their inability to grow in fresh growth medium.

### 4.7. Aflatoxin Quantification

Qualitative comparisons of aflatoxin accumulation in the growth media were performed using thin-layer chromatography (TLC) as described previously [54]. Quantification of aflatoxin for the aflatoxin uptake experiments was performed using a Veratox for Aflatoxin ELISA kit (Neogen Food Safety, Lansing, MI, USA) and measured on a Stat Fax 4700 Microstrip Reader (Awareness Technologies, Palm City, FL, USA).

### 4.8. Statistical Analyses

Statistical analyses for this study were conducted using the GraphPad Prism Software (GraphPad, La Jolla, CA, USA). The statistical significance of two-tailed *p*-values were determined using an unpaired *t*-test, using *n* = 3 and *p* < 0.05. For the gene expression studies, a two-fold increase or decrease in transcript level was our cutoff for comparing expressions between two genes.

## Figures and Tables

**Table 1:** Amino acid sequences of the SODs analyzed in the study. The names of the SODs as annotated in the gene bank database and their accession numbers are mentioned above each sequence within the shaded rows.

**FeSOD** **(Gene bank accession number EED58116.1)**
MLPRFLRPQSTLRAVSSLTQKPASALPRFQTRGLHRVPQLTHDTHFKNNGIQELLSPEAFDFAWTQYQTLLIDKLNLLTQDTVDADAKPGELLVKYSRRPEMASVFNYASMAHNNHFFFNCLSPTPTQIPDKFAKDIVDTCSSIESLKLDFLATANAMFGPGFVWL AKNLEREGLMHIFCTYNAGSPYPAAHSRRQPVDMATHSPDAPLGNQFAGAMGAHSANQKSLAPGAVDVQPILCVNTWEHVWMMDYGIGGKAEYLERWWDRINWEVVFDNYNAVS SMKGTRHAANRNRSLSML
**FeSODA** **(Gene bank accession number EED55486.1)**
MAASLIRTSARTALRAGASATPKAAGVAGLTFARGKATLPDLAYDYGALEPSISGKIMELHHKNHHQTYVNSYNTAIEQLQEAVAKEDITTQINLKPLINFHGGGHINHTLFWENLAPKSQGGGEPPSGALAKAIDESFGSLGEFQSKMNAALAGIQGSGWAWLVK DKQTGNIGIKTYAVSSSLTRTLSLVSSSLFSVLMLGSTPTTFNTRTARLSTSAPSGTSSTGRRLRSASRKRAKVGWITCSRSIPAGSGKLIFLVWDPLRPLRIFFPHLTSQAINSLEMSAESPGEKRGGFRAFFAGALRPKKSRQVLRKASTPNLKEGLQSKDDVPA MPSLTPLEAHRLKYREVNLQKDTQLGETHDHTAMLHSIGVGELDPSDPHAQLHEFDNRPPGEPMIASLTSDLWAKVTEYLNPAERASLAFSSRTLYARLGREPWITINLPENHDYKADFLISQDRLLPHHLLCFPCGKYHRRTQEGYEKLQPADIINPLFDCPNARNN ALPAPRHRITHGRVLYFTFHQLVMRAYRFGPRYGISADSLSRRWRRDGWSHQTRYHIHQGRLLMRVVSTCFAEPGLSASQQRLLLYSRDDYWPYFSVCAHWRDGELMNVCKCALGHIPVPRTTNGLQGLEHRAKDMYHRREHNPNALASLCGKCRPMRRCPECPSEYL VEVKLTEDRSGSHRNLFRHAIVVTRWSDLGDGRSPRLSKEWAAINGDEAGEGYDSFEKIGKRAISGIFESAITDDTLPGQRILSMNPKERSWVRLGIIGIEVPYLYFALGVICGGKLGVLSGVIFCIILYYTRVGVWVGWVGWVGWVGWVGWVGWVGWVGWIGLCGFI
**CuZnSOD1** **(Gene bank accession number EED46237.1)**
MVKAVAVLRGDSKISGTVTFEQADANAPTTVSWNITGHDANAERAFHVHQFGDNTNGCTSAGPHFNPFGKEHGAPEDENRHVGDLGNFKTDAEGNAVGSKQDKLIKLIGAESVLGRTLVIHAGTDDLGRSEHPESKKTGNAGARPACGVIGIAA
**Cytosolic CuZn SOD** **(Gene bank accession number EED49986.1)**
MLTKSLFAGAALGLSLSSAVAHEAPVVEGNEPQTVYEAVLQDKDNTTVRGTFTTHGAEDGIGIQFRVALTGVPKDTFLNYHIHDNPVPKDGNCYATGGHLDPYKRGDQPPCNTTVPQTCQVGDISGKHGPVWTADGNFEVLYRDFFLSNVEDTIAFFGNRSVVV HLPDNKRINCGNFHLVSDGEEKKKKEEAKEDQGC
**MnSOD** **(Gene bank accession number EED56070.1)**
MATTFSLPPLPYAYDALEPVICKQIMEIHHQKHHQTYITNLNAALSAQSTALAANNIPQLINLQQKIKFNGGGHINHSLFWKNLAPHASPETNIDQAAPVLKAAIEAQYGSVEKFKEAFGATLLGLQGSGWGWLVANGPGGKLEIVSTKDQDPVTDKVPVFGVD MWEHAYYLQYFNNKASYVEGIWKVLNWRTAEDRFKNGVEGSALLKL

**Table:** (**A**) Presence of signatures of conserved domains.

Gene	Conserved Domain	Conserved Sequence
FeSOD	None	None
FeSOD A	None	None
CuZnSOD1	CuZn superoxide dismutase signature 1	AFHVHQfGDnT
	CuZn superoxide dismutase signature 2	GNAGaRpACgvI
CuZnSOD cytosolic	None	None
MnSOD	Manganese and iron superoxide dismutases signature	DmWEHAYY

**Table:** (**B**) Analysis of SOD frequency patterns in FeSOD, FeSOD A and CuZnSOD cytosolic.

**(i). Frequency data for the presence of frequency patterns in the 390 SODs within the PROSITE database**							
**ID**	**Patterns**		**Sites corresponding to the Patterns**			**Frequency**	
PS00001	ASN_GLYCOSYLATION (pattern)		*N*-glycosylation site			302	
PS00004	CAMP_PHOSPHO_SITE (pattern)		cAMP- and cGMP-dependent protein kinase phosphorylation site			47	
PS00005	PKC_PHOSPHO_SITE (pattern)		Protein kinase C phosphorylation site			273	
PS00006	CK2_PHOSPHO_SITE (pattern)		Casein kinase II phosphorylation site			331	
PS00007	TYR_PHOSPHO_SITE (pattern)		Tyrosine kinase phosphorylation site			37	
PS00008	MYRISTYL (pattern)		N-myristoylation site			387	
PS00009	AMIDATION (pattern)		Amidation site			35	
PS00016	RGD (pattern)		Cell attachment sequence			38	
PS00017	ATP_GTP_A (pattern)		ATP/GTP-binding site motif A (P-loop)			14	
PS00342	MICROBODIES_CTER (pattern)		Microbodies C-terminal targeting signal			34	
PS50310	ALA_RICH (profile)		Alanine-rich region profile			2	
PS50321	ASN_RICH (profile)		Asparagine-rich region profile			1	
PS50324	SER_RICH (profile)		Serine-rich region profile			1	
**(ii).** **Frequency of occurrence of the most frequent patterns in FeSOD, FeSOD A and CuZnSOD cytosolic**							
**4 most frequent sites in SODs (from table above)**		**Consensus Pattern**		**Frequency of occurrence in**			
				**CuZnSOD** **cytosolic**	**FeSOD**		**FeSODA**
N-myristoylation site		G-{EDRKHPFYW}-x(2) -[STAGCN]-{P}		5	4		15
CK-2 phosphorylation site		[ST]-x(2)-[DE]		3	1		7
*N*-glycosylation site		N-{P}-[ST]-{P}		3	1		1
Protein kinase C phosphorylation site		[ST]-x-[RK]		2	6		11

**Table 3:** List of primers used in the study.

NO.	Genes	Primer Sequences
1	*nor-1*	F 5′-CACTTAGCCAGCACGATCAA-3′
		R 5′-ATGATCATCCGACTGCCTTC-3′
2	*ver-1*	F 5′-AACACTCGTGGCCAGTTCTT-3’
		R 5′-ATATACTCCCGCGACACAGC-3’
3	*β-tubulin*	F 5′-TCTCCAAGATCCGTGAGGAG-3′
		R 5′-TTCAGGTCACCGTAAGAGGG- 3′
4	FeSOD	F 5′-GAGATGGCCTCCGTATTCAA-3′
		R 5′-CATCAATCCTTCCCTCTCCA-3′
5	FeSOD A	F 5′-CCAAGGAGGACATCACCACT-3′
		R 5′-GCATAGGTCTTGATGCCGAT-3′
6	CuZnSOD1	F 5′-CACCAGTTCGGTGACAACAC-3′
		R 5′-GTGTTCACTACGGCCAAGGT-3′
7	CuZnSOD cytosolic	F 5′-CCTCCTTGCAATACAACCGT-3′
		R 5′-GTCTTCCTTCGCCTCTTCCT-3′
8	MnSOD	F 5′-CCACATCAACCACTCCCTCT-3′
		R 5′-TCCTGATCCTTCGTCGAAAC-3′

## Supplementary Materials

The following are available online at http://www.mdpi.com/2072-6651/10/2/57/s1, Figure S1: Raw expression data of the SOD genes in SU-1 and AFS10.
