# Supplementary material for: Activation of Aflatoxin Biosynthesis Alleviates Total ROS in Aspergillus parasiticus
Source: Toxins (Basel). 2018 Jan 29;10(2):57. doi: 10.3390/toxins10020057 (PMC5848158; doi:10.3390/toxins10020057)
Supplement: Supplementary file 1 [file toxins-10-00057-s001.pdf]

Supplementary Materials: Activation of Aflatoxin Biosynthesis Alleviates Total ROS in *Aspergillus parasiticus*

Gabriel J. Kenne, Phani M. Gummadidala, Mayomi H. Omebeyinje, Ananda M. Mondal, Dominic K. Bett, Sandra McFadden, Sydney Bromfield, Nora Banaszek, Michelle Velez-Martinez, Chandrani Mitra, Isabelle Mikell, Saurabh Chatterjee, Josephine Wee and Anindya Chanda

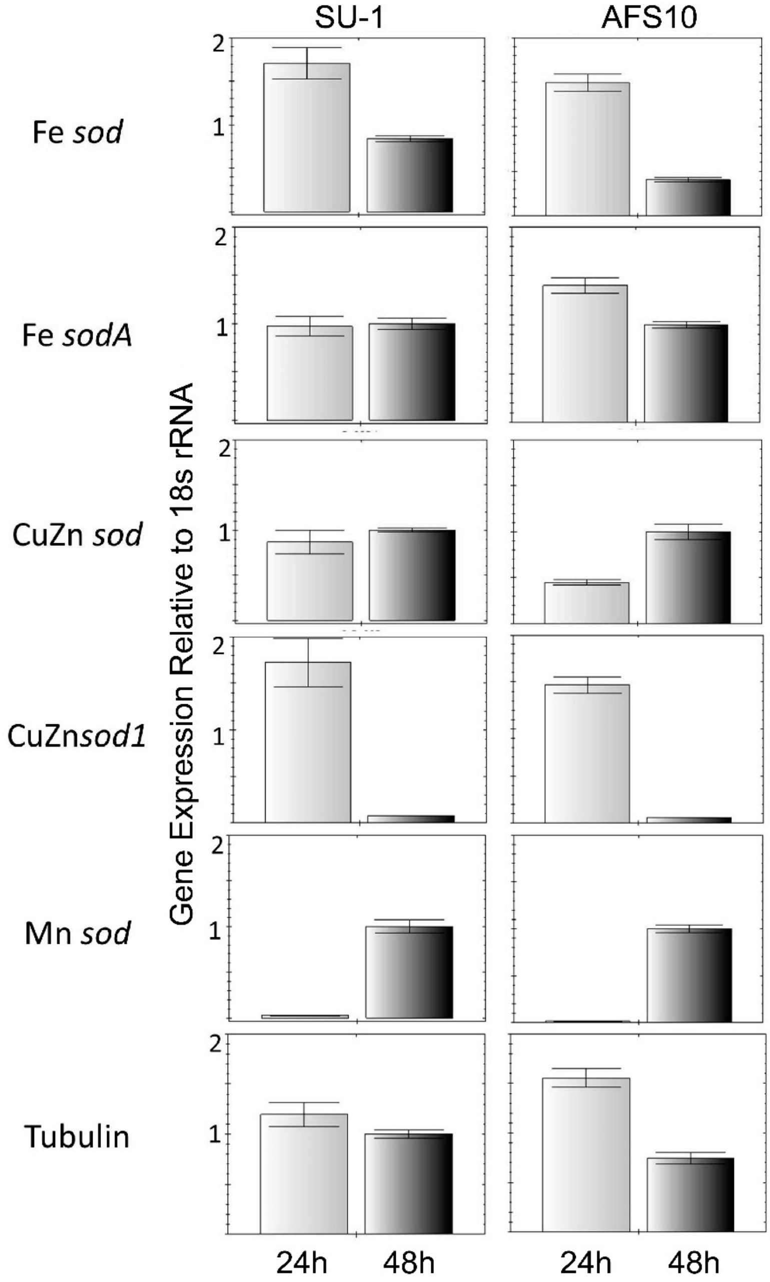

Figure S1. Raw expression data of the SOD genes in SU-1 and AFS10. qPCR comparison of SOD gene expression in the two strains at 24 h and 48 h of culture growth. All expression quantifications were conducted in triplicate. For each gene the expression value was normalized against and 18s rRNA reference gene and compared to a β-tubulin control.
